# Antioxidant, Anti-Inflammatory and Antithrombotic Effects of Ginsenoside Compound K Enriched Extract Derived from Ginseng Sprouts

**DOI:** 10.3390/molecules26134102

**Published:** 2021-07-05

**Authors:** In-Hee Baik, Kyung-Hee Kim, Kyung-Ae Lee

**Affiliations:** 1 R&D Center, Youngjin Bio Co., Suwon 16614, Korea; yjbio21@naver.com; 2 Department of Food and Nutrition, Duksung Women’s University, Seoul 01370, Korea; khkim@duksung.ac.kr; 3 Department of Food and Nutrition, Anyang University, Anyang 14028, Korea

**Keywords:** ginseng sprout, ginsenoside source, compound K, enzymatic conversion, antioxidant, anti-inflammatory, antithrombotic

## Abstract

Partially purified ginsenoside extract (PGE) and compound K enriched extract (CKE) were prepared from ginseng sprouts, and their antioxidant, anti-inflammatory and antithrombotic effects were investigated. Compared to the 6-year-old ginseng roots, ginseng sprouts were found to have a higher content of phenolic compounds, saponin and protopanaxadiol-type ginsenoside by about 56%, 36% and 43%, respectively. PGE was prepared using a macroporous adsorption resin, and compound K(CK) was converted and enriched from the PGE by enzymatic hydrolysis with a conversion rate of 75%. PGE showed higher effects than CKE on radical scavenging activity in antioxidant assays. On the other hand, CKE reduced nitric oxide levels more effectively than PGE in RAW 264.7 cells. CKE also reduced pro-inflammatory cytokines, such as tumor necrosis factor-α, interleukin (IL)-1β and IL-6 than PGE. Tail bleeding time and volume were investigated after administration of CKE at 70–150 mg/kg/day to mice. CKE administered group showed a significant increase or increased tendency in bleeding time than the control group. Bleeding volume in the CKE group increased than the control group, but not as much as in the aspirin group. In conclusion, ginseng sprouts could be an efficient source of ginsenoside, and CKE converted from the ginsenosides showed antioxidant, anti-inflammatory and antithrombotic effects. However, it was estimated that the CKE might play an essential role in anti-inflammatory effects rather than antioxidant effects.

## 1. Introduction

Ginseng (*Panax ginseng* Meyer) is a perennial herbaceous plant that has been used for a long time in Asian countries such as Korea and China for its various medicinal properties. It has also been developed as processed foods and pharmaceuticals in Eastern countries and Western countries [1,2]. In general, ginseng roots grown for 4–6 years are preferred and used for medicinal prescriptions or health foods in the market [3]. However, it does not seem easy to cultivate ginseng for years without agricultural chemicals such as pesticides because such slow-growing plants can be susceptible to various diseases and pathogens during the cultivation [4]. Currently, there is a growing interest in pesticide-free ginseng resources. For this purpose, ginseng cultivated in an organic agriculture system has been produced, but it is not widely used because of its much higher price than generally available ginseng products. As an alternative, various hydroponic or greenhouse cultivation systems have been applied to ginseng cultivation to provide clean and inexpensive raw materials.

Ginseng sprouts are the young ginseng grown from seedlings for weeks to months in hydroponic or artificial soil cultivation systems. They have been developed as medicinal vegetables or foods due to their relatively short period growth in a soil-less cultivation system without pesticides. The composition and efficacy of the ginseng sprouts have been studied extensively. Lee et al. [5] investigated the ginsenoside composition and phenolic compound content in a hydroponic-culture of ginseng, and reported an increase of anti-oxidative activity. Kim et al. [6] studied changes of growth and ginsenoside contents of ginseng cultured in an aeroponic system with some natural additives. Seong et al. [7] reported the changes in growth, active ingredients, and rheological properties of greenhouse-cultivated ginseng. Changes in ginsenoside content depending on growth period and harvest time were investigated in different parts of ginseng sprouts [8]. It was reported that the cultivation of ginseng sprouts with plasma-treated water increased amino acid and ginsenoside content [9].

However, most studies have been conducted on the intact compounds contained in ginseng sprouts and their efficacies, and little information is available on the processing of ginseng sprouts or efficacies of the processed products. Kim et al. [10] reported that ginsenoside content decreased whereas total phenolic and flavonoid content increased during the roasting process of ginseng sprouts. Hwang et al. [11] investigated the improved antioxidant, anti-inflammatory, and anti-adipogenic properties of hydroponic ginseng fermented by *Leuconostoc mesenteroides*. More studies on the application and efficacies are required for the wide use of ginseng sprouts because ginseng sprouts are used mainly as decorative foods in restaurants or personal favorite foods rather than medicinal vegetables in the market. In this this study, we investigated whether ginseng sprouts could be an efficient clean source of ginsenosides such as compound K (CK) and whether the processed, rare ginsenoside derived from the ginsenosides could have beneficial health effects.

## 2. Results

### 2.1. Content of Phenolic Compounds, Crude Saponin and Ginsenoside

Total phenolic compounds and crude saponin content of ginseng sprouts and 6-year ginseng roots were compared as shown in Table 1. Leaves of ginseng sprouts showed the highest phenolic compound content, about 5 times higher than that of stems and 4.3 times higher than roots of ginseng sprouts. Leaves of ginseng sprouts showed the highest saponin content, about 7.2 times higher than that of stems and 2.8 times higher than roots of ginseng sprouts. However, the ratio of leaves to the total dry weight of whole ginseng sprout was relatively low compared to roots. The total phenolic compounds and saponin of the whole ginseng sprout was 10.20 ± 1.51 mg/g and 15.89 ± 0.71%, which was about 56% and 36% higher, respectively than that of 6-year-old ginseng.

Protopanaxadiol (PPD) type ginsenosides, which could be converted to CK were measured in ginseng sprouts. The contents of PPD type ginsenosides, depending on the part, showed a similar tendency to saponin (Table 2). The highest ginsenoside content was obtained in the leaves, which was more than 4 times higher than that of roots or stems of ginseng sprout. Total PPD type ginsenoside of the whole ginseng sprout was 16.64 mg/g, which was about 43% higher than that of a 6-year ginseng root.

### 2.2. Enzymatic Conversion of PPD Type Ginsenosides to CK

#### 2.2.1. Preparation of CKE

A commercial enzyme, Plantase AK from a fungal origin, showed high activity for hydrolysis of the diverse glycosides of ginsenosides. The changes of ginsenosides during the reaction of the optimized condition were investigated, as shown in Table 3. Ginsenoside Rb1 and Rb2 were rapidly converted to Rd or F2 while conversion of Rc was relatively slow. Ginsenoside Rd and F2, the intermediates of conversion to CK, increased in the middle of reaction and most of them converted to CK at the end of the reaction, but Rd seemed to convert to CK more efficiently than F2. In addition to CK, a small amount of Rg3 was also produced. The CK conversion rate from PPD type ginsenoside was about 75%, calculated as a protopanaxadiol equivalent.

#### 2.2.2. Mass Spectral Fragmentation of CK

**Figure 1:**
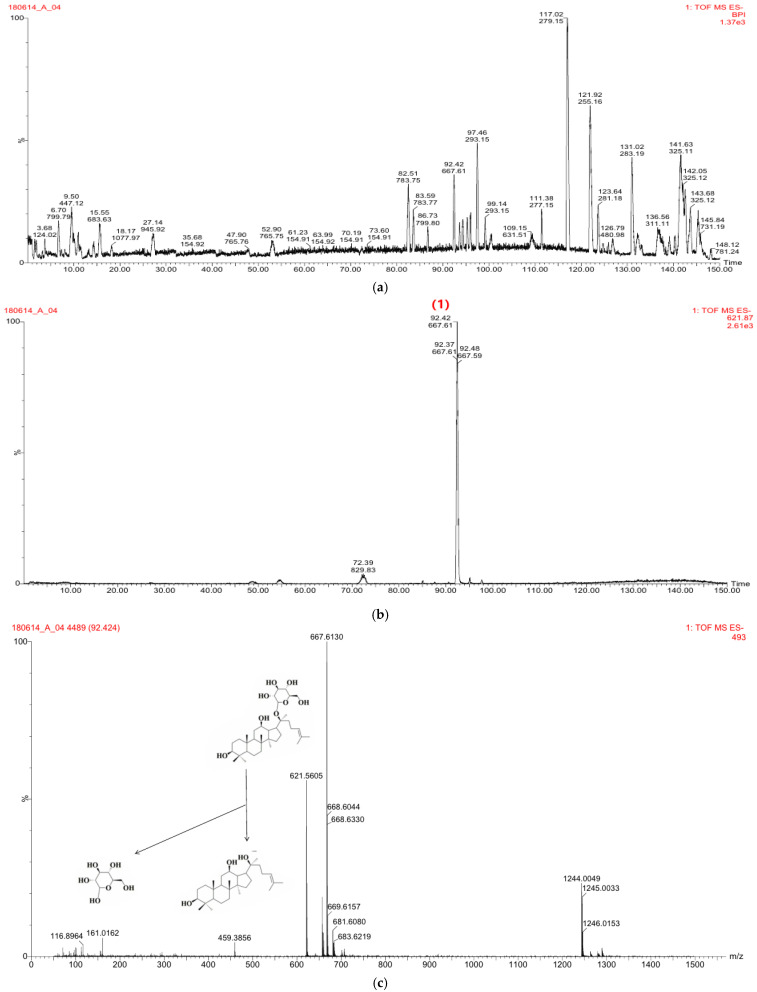
Identification of CK by LC-MS analysis. (**a**) base peak intensity chromatograms of ESI-MS of reactant during enzymatic conversion, (**b**) base peak chromatograms of CK at 92.42 min, (**c**) Structure and mass spectrum of CK derived from ginseng sprout.

The CK obtained by the enzyme reaction was identified by LC-MS analysis (Figure 1a–c). CK eluted at 92.42 min under the conditions described. Negative ion mode was applied for data acquisition because CK showed in-source dissociation in positive ion mode. The protonated molecular ion of CK at *m*/*z* 621.5605 produced fragment ions at m/z 459.3856 and *m*/*z* 161.0162 by losing a glucose moiety. An [M + HCOO^−^] ion at *m*/*z* 667.6130 was also observed.

### 2.3. Biological Effects of Compound K Enriched Extract (CKE)

#### 2.3.1. Antioxidant Activity of Partially Purified Ginsenoside Extract (PGE) and CKE

**Figure 2:**
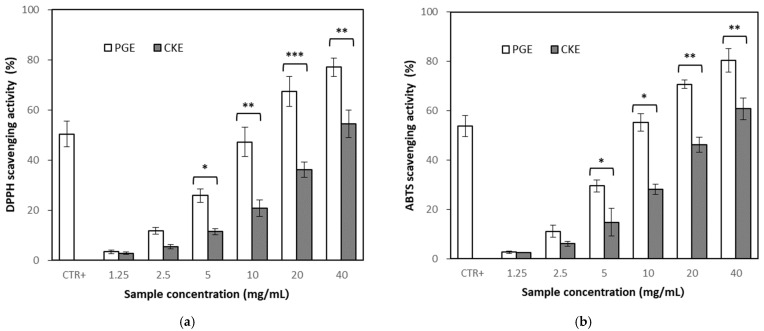
Antioxidant effects of partially purified ginsenoside extract (PGE) and compound K enriched extract (CKE). Result expressed as a mean ± SD (*n* = 3). (**a**) on 1,1-diphenyl-2-picrylhydrazyl (DPPH) scavenging assay, (**b**) 2,2′-azino-*bis*-3-ethylbenzthiazoline-6-sulphonic acid (ABTS) scavenging assay. CTR+, Positive control (ascorbic acid, 0.10 ± 0.01 mg/mL). An asterisk indicates a significant difference between the PGE and CKE at each concentration (*, *p* < 0.05; **, *p* < 0.01; ***, *p* < 0.001).

Antioxidant activity was evaluated by 1,1-diphenyl-2-picrylhydrazyl (DPPH) assay and 2,2′-azino-*bis*-3-ethylbenzthiazoline-6-sulphonic acid (ABTS) assay. Measurement of DPPH radical scavenging activity is the direct estimate method of antioxidation activity. Treatment of PGE and CKE showed increased DPPH radical scavenging activity in a concentration-dependent manner (Figure 2a), but PGE showed higher effects than CKE. Median scavenging concentration (SC_50_), which showed 50% of scavenging, was identified as 10.09 ± 0.06 mg/mL for PGE and 36.43 ± 0.05 mg/mL for CKE, respectively, while SC_50_ of ascorbic acid as a positive control was 0.10 ± 0.01 mg/mL.

Radical scavenging effect was also confirmed by ABTS assay, which was more stable at pH change. The ABTS assay resulted in a similar effect with DPPH assay, showing higher effects of PGE than CKE (Figure 2b).

#### 2.3.2. Effects of PGE and CKE on Cell Viability

**Figure 3:**
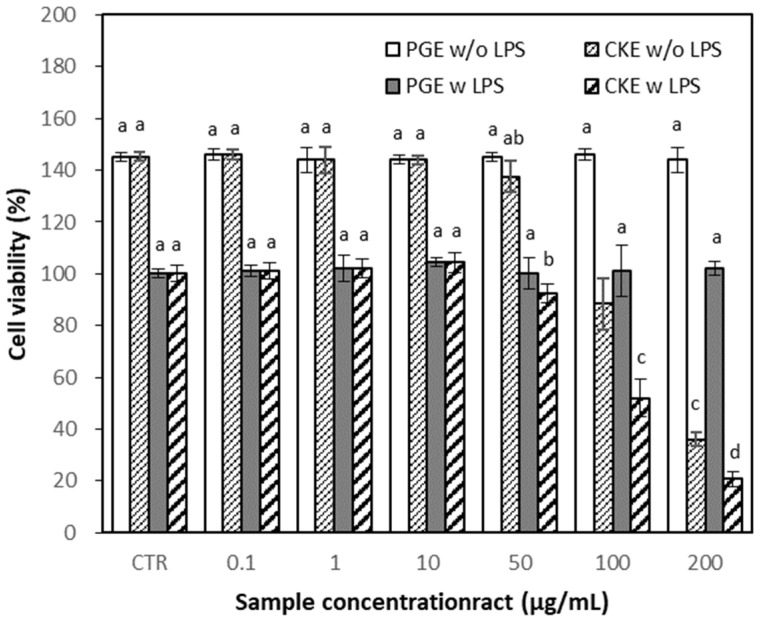
Effect of PGE and CKE on cell viability. RAW 264.7 cells were treated with PGE or CKE for 3 h and incubated with or without LPS for 24 h. CTR, Control (with or without LPS 1 μg/mL). Result is expressed as a mean ± SD (*n* = 3). Alphabet notation represents a significant difference among samples based on Duncan’s multiple range test (*p* < 0.05).

Cytotoxic effects of PGE and CKE were assessed based on 3-(4,5-dimethylthiazol-2-yl)-2,5-diphenyltetrazolium bromide (MTT) assay before evaluating their anti-inflammatory effects. MTT assays were performed using the RAW 264.7 cells treated with different concentrations of PGE and CKE in the presence or absence of lipopolysaccharide (LPS, 1 μg/mL), to exclude the possibility that the inhibition of nitric oxide (NO) production was due to cytotoxicity caused by PGE or CKE. PGE did not affect cell viability at the tested concentration up to 200 μg/mL, while CKE showed a pronounced cytotoxic effect at 100 and 200 μg/mL in both with and without LPS (Figure 3). Based on these results, the rest of the experiments were carried out at concentrations of 0.1–50 μg/mL.

#### 2.3.3. Effects of PGE and CKE on NO Levels

**Figure 4:**
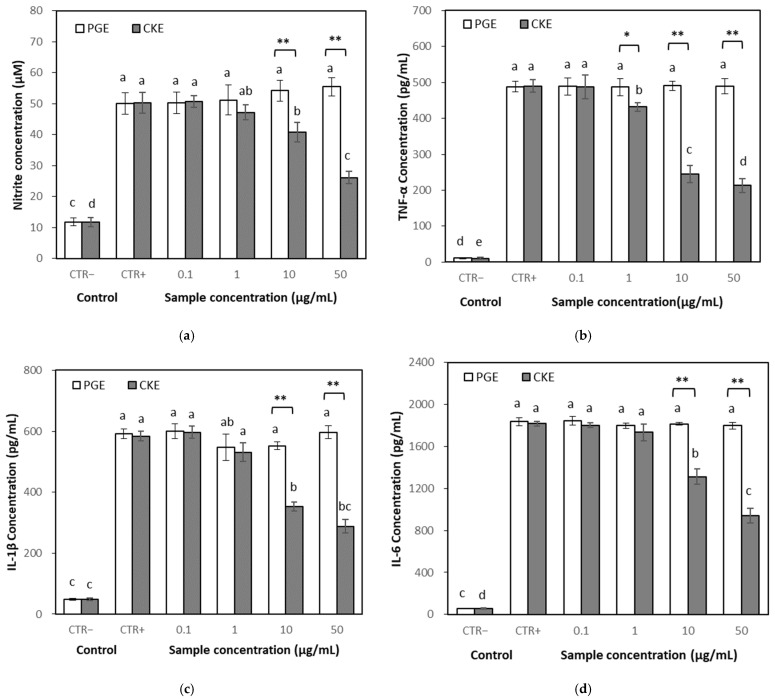
Effect of PGE and CKE extract on the production of nitric oxide (NO), tumor necrosis factor-α (TNF-α), Interleukine (IL)-1β and IL-6. RAW 264.7 cells were cultured with different concentrations of PGE or CKE for 24 h with LPS (1 μg/mL). CTR−, Negative control. CTR+, Positive control (LPS 1 μg/mL). Result expressed as a mean ± SD (*n* = 3). (**a**) Effect on NO; (**b**) Effect on TNF-α; (**c**) Effect on IL-1β; (**d**) Effect on IL-6. Different letters above the bars of the same concentration indicate significant differences among treatment means (*, *p* < 0.05; **, *p* < 0.01).

NO is one of the most important pro-inflammatory mediators [12,13]. The effect of the CKE on the NO production was determined in LPS-stimulated RAW 264.7 cells. Treatment of the cells with LPS alone markedly induced NO production compared to the untreated control. However, co-treatment with CKE significantly suppressed NO production at over 10 μg/mL, while that with PGE did not show an effect up to 50 μg/mL (Figure 4a).

#### 2.3.4. Effects on Inflammatory Cytokines

Levels of pro-inflammatory cytokines such as tumor necrosis factor-α (TNF-α), Interleukine (IL)-1β and IL-6 were quantified in the culture medium of RAW 264.7 cells using enzyme-linked immunosorbent assay (ELISA) to identify the anti-inflammatory properties of CKE. The production of TNF-α, IL-1β and IL-6 was markedly increased by LPS stimulation. Co-treatment with CKE significantly decreased cytokine production, while PGE treatment did not show an effect at the same concentration (Figure 4b–d).

#### 2.3.5. Bleeding Time and Volume

**Figure 5:**
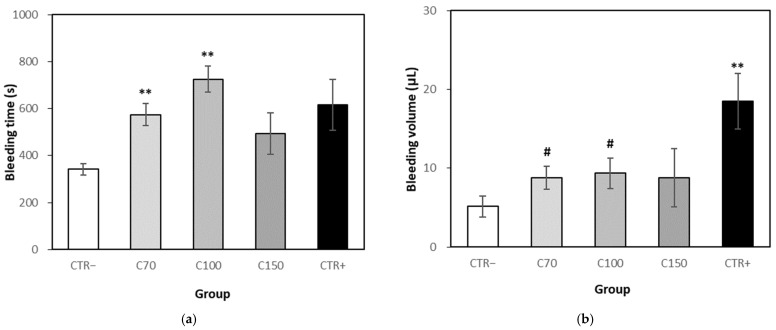
Effect of CKE on tail bleeding time and volume. The test samples were administered orally once a day for 7 days. Result expressed as a mean ± SEM. (**a**) Effect on the bleeding time, (**b**) Effect on the bleeding volume). ** represents *p* < 0.01 compared to CTR- group; # represents *p* < 0.05 compared to CTR+ group. Abbreviation: CTR−, Vehicle control (distilled water), *n* = 6; C70, CKE 70 mg/kg/day, *n* = 6; C100, CKE 100 mg/kg/day, *n* = 6; C150. CKE 150 mg/kg/day, *n* = 6; CTR+, Positive control (aspirin 100 mg/kg/ day), *n* = 6.

During the test period of the bleeding assay, no death of mice and no unusual symptom was observed, and the bodyweight of the test and positive control group did not show significant differences. The tail bleeding time of test group 70 and 100 mg/kg/day showed a significant increase in bleeding time compared to the control group (*p* < 0.01). The bleeding time of the test group 150 mg/kg/day increased 43.99%, but there was no statistical difference compared to the control group. The bleeding time of the aspirin group showed a 79.81% increase compared to the control group, but it did not show a statistical difference with the test group (Figure 5a).

The test groups at 70, 100 and 150 mg/kg/day showed an increased blood volume by 70.40%, 81.54% and 70.76%, respectively, compared to the control group. The bleeding volume of the aspirin group showed a significant difference to control. However, the bleeding volume of 70 and 100 mg/kg/day in the test group showed a statistically significant reduction compared to the aspirin group. There was no statistical difference between the 150 mg/kg/day group and the aspirin group, but a bleeding reduction of about 52.30% was observed (Figure 5b).

## 3. Discussion

This study was conducted to investigate whether ginseng sprouts could be an efficient, clean ginsenoside source for a rare ginsenoside CK, and whether the CK containing extract could be used to prevent or treat inflammation and blood coagulation.

CK (20-*O*-β-d-glucopyranosyl-20(*S*)-protopanaxadiol) is a minor tetracyclic triterpenoid which is either absent in natural ginseng or available at very low concentrations [14]. It is a hydrolyzed metabolite absorbed and found in organs or blood after oral ingestion of PPD type ginsenosides [15]. Akao et al. [16] investigated the absorption of CK in rat plasma, and Lee et al. [17] demonstrated the absorption, distribution, and metabolism of CK in humans. CK is one of the rare ginsenosides, and it is of great interest due to its diverse biological activities and high pharmacological activities [18,19]. However, the minute amount of CK in ginseng cannot satisfy the needs of commercial purposes [20]. Therefore, it is important to develop useful methods for the mass production of the rare ginsenoside. Many efforts were conducted to provide CK efficiently and at low cost. [21,22,23]. It was reported that the ginseng leaves contained much higher ginsenoside content compared to ginseng roots [24,25], but they are limited in their use because ordinary ginseng is harvested 1–2 times a year in general. On the other hand, ginseng sprouts, with a higher ginsenoside content than ginseng roots, can be cultivated and harvested year-round. Regarding the considerable amounts of abnormal size and shape that appear during the cultivation of ginseng sprouts, which are not available for the market, the abnormal products can be used as raw materials for ginsenosides or processed products. Since the PPD type ginsenoside content of ginseng sprouts was much higher than that of 6-year-old ginseng roots, CK was provided effectively by an enzymatic hydrolysis from the ginsenosides. Therefore, ginseng sprouts are considered as an efficient, clean source of ginsenosides than ginseng roots.

In this study, the anti-inflammatory and antithrombotic effects were investigated to evaluate the beneficial effects of CK.

Antioxidant activity, which is closely related to anti-inflammatory and antithrombotic effects, was determined preliminary to anti-inflammatory activity. The concentrated ginsenoside complex, PGE, showed higher activities than the CK enriched fraction, CKE, in both DPPH and ABTS radical scavenging assay. PGE contained more than 90% of the extracted phenolic compounds, whereas CKE contained a relatively low level (about 27%) of phenolic compounds. The difference in antioxidant activity was presumed to be due to the difference in phenolic compounds between PGE and CKE. It was estimated that the CK might not be essential in antioxidant activity than phenolic compounds.

Inflammation is an essential biological response to restore tissue homeostasis. However, immoderate inflammation may result in devastating impacts by causing unnecessary collateral damage [26]. Various studies have been conducted to understand the pharmacological mechanisms of ginseng and ginsenosides for reducing inflammation. It was reported that CK exhibited anti-inflammatory effects mainly by reducing inflammatory mediators, but its efficacies may vary depending on its preparation method, content and composition of extract or solubility system [27]. In this study, CKE derived from ginseng sprouts showed reducing effects in a dose-dependent manner on NO and pro-inflammatory cytokines, such as TNF-α, IL-1β and IL-6 in RAW 264.7 cells. CKE showed higher anti-inflammation effects than PGE in RAW 264.7 cells, while PGE showed higher antioxidant activity than CKE in antioxidant assays. It was presumed that the high content of CK in the CKE might play an essential role in anti-inflammatory effects.

In vivo, blood clotting and dissolution are always in equilibrium, and blood clots do not form during normal circulation. However, if the balance is broken due to various causes such as inflammation, blood clots occur and blood vessels are blocked, blood circulation is disturbed and the supply of nutrients and oxygen to the tissues is stopped. It has been studied in vitro and in vivo that inflammation and blood clotting are closely related, that is, inflammation can beget local thrombosis and thrombosis can amplify inflammation [28,29]. The pro-inflammatory cytokines such as TNF-α were reported to be upregulated in most inflammatory conditions and contribute to change in blood coagulation inducing complement 3 and platelet activation [30,31,32]. Ginseng and various ginsenosides, such as Rg1, Rg3, Rp3 and F4, have been studied to effectively inhibit platelet activation and thrombosis, even though there are some controversial reports [33,34]. CKE from ginseng sprouts showed antioxidant and anti-inflammatory effects, which indicate its potential for antithrombotic effect. Platelet aggregation and blood coagulation tests are widely used as an assessment of hemostatic action for platelets or anti-thrombotic effect of drugs in vitro and in vivo [35,36]. In this study, a bleeding assay was conducted to estimate the antithrombotic effect of CKE, because in vivo blood coagulation and in vitro platelet aggregation may show different results depending on test materials and condition. Treatment of CKE was considered to reduce blood coagulation, because a significant increase or increased tendency of bleeding time, and a tendency to increase bleeding volume were observed compared to the control group. The positive control, aspirin, also increased bleeding time and volume compared to the vehicle control group. However, a significant decrease or decreased tendency in bleeding volume was observed between the CKE group and the aspirin group, while there was no significant difference in bleeding time between them. Aspirin has been used as an anti-inflammatory and anti-platelet drug, to prevent the risk of cardiovascular disease, but chronic use of the drug may result in another risk such as excess bleeding [37,38]. The CKE showed increased effects on both bleeding time and bleeding volume, but the increase of bleeding volume was not as much as in the aspirin group. This effect could be an advantage in treating thrombosis because excessive bleeding is undesirable. Further investigation for the effect of CKE on blood coagulation and platelet aggregation will be performed.

## 4. Materials and Methods

### 4.1. Plant Materials and Extraction

Medium-sized ginseng sprouts (total height of 15–20 cm) were purchased in Yangpyeong, Gyeonggi-do, Korea, in March 2017. The ginseng sprouts were washed with tap water and soaked in distilled water. Leaves, stems and roots were separated and dried in a drying oven at below 50 °C. The dried samples were ground into coarse powder and stored at −20 °C. Six-year-old ginseng roots were purchased in Poonggi, Gyeongsangbuk-do, Korea and samples for extraction were prepared in the same method as ginseng sprouts. For the extraction of saponin or ginsenoside, samples were extracted with 10 volumes of 70% (*v*/*v*) ethanol for 16 h at 40 °C and the extract was evaporated at 40 °C in a rotary evaporator.

### 4.2. Chemicals

Ginsenoside Rb1, Rb2, Rc, Rd, F2, Re, Rg3 and CK were purchased from Ambo Institute (Daejeon, Korea), and other analytical reagents were purchased from Sigma-Aldrich (St. Louis, MO, USA). LPS, 3-(4,5-dimethylthiazol-2-yl)-2,5-diphenyltetrazolium bromide (MTT), dimethyl sulfoxide (DMSO) were purchased from Sigma Chemical Co. (St. Louis, MO, USA). Dulbecco’s modified eagle’s medium (DMEM) and fetal bovine serum (FBS) were purchased from Hyclone (Logan, UT, USA) and penicillin/streptomycin solution was a product of Thermo Fisher Scientific (Waltham, MA, USA). NO detection kit was purchased from iNtron (Seongnam, Korea). ELISA kits of TNF-α, IL-1β and IL-6 were purchased from Thermo Fisher Scientific.

### 4.3. Preparation of PGE and CKE

To increase the enzymatic CK conversion efficiency, it is preferable that the extract contains fewer impurities and has a high ginsenoside content. PGE was prepared using an adsorption column filled with a macroporous adsorption resin Amberlite XAD-7 (Dow Chemical, USA) before enzymatic hydrolysis. The crude extract was loaded onto the column, washed with water and subsequently 10% (*v*/*v*) ethanol, and then eluted with 70% (*v*/*v*) ethanol. Ginsenoside content of the eluted fraction was concentrated about 3 times compared to before purification. The eluent was evaporated and concentrated at 40 °C in a rotary evaporator. To prepare CKE from PGE, a commercial enzyme, Plantase AK (Bision Biochem. Corp., Seoul, Korea) was tested at various temperatures, pH, time and enzyme concentration to optimize the reaction. The optimized conditions for CK conversion from ginsenosides were; enzyme concentration 200 mg/g solid, temperature 40 °C, pH 5.0, and reaction time 48 h. After the enzyme reaction, the precipitate was collected by centrifugation at 3000 rpm. The obtained precipitate was solubilized in absolute ethanol and filtered through 0.2 μm membrane filter. The filtrate was concentrated again and dispersed in water, and the precipitate was collected as CKE. The conversion rate of CK from PPD type ginsenosides was calculated as the ratio of PPD equivalent, that is, PPD content of CK after enzyme reaction to the sum of PPD content of Rb1, Rb2, Rc, Rd and F2 before enzyme reaction.

$$\mathrm{Conversion}\;\mathrm{rate}\;\left(\%\right)=\frac{\left(\mathrm{Compound}\;K\;\mathrm{content}\times 0.74\right)}{\left(\mathrm{Rb}1\times 0.41\right)+\left(\mathrm{Rb}2\times 0.42\right)+\left(\mathrm{Rc}\times 0.42\right)+\left(\mathrm{Rd}\times 0.48\right)+\left(F2\times 0.58\right)\;}\times 100$$

### 4.4. Analysis of Phenolic Compound, Saponin and Ginsenoside

The content of total phenolic compounds was determined by the method of Folin and Denis [39], using gallic acid as a standard (mg gallic acid equivalents). The total crude saponin content of ginseng sprouts and 6-year ginseng roots was determined by the method of Hiai et al. [40] with slight modification. The composition of ginsenosides in samples was measured using HPLC (Hitachi Chromaster 5110, Japan). A reverse-phase column (Optimapak C18, 4.6 × 250 mm) was used and the mobile phase was a binary eluent of A (%, water) and B (%, acetonitrile) under the following gradient system: 0 min (80:20), 10 min (80:20), 25 min (76:24), 30 min (67:33), 42 min (63:37), 57 min (20:80), 60 min (0:100). 65 min (40:60), 70 min (80:20%). The flow rate was 1.0 mL/min, injection volume was 10 μL and ginsenosides were detected at 203 nm.

CK formed by enzyme reaction was identified by LC-MS analysis. The analysis was performed using an HPLC system (LC-20AD, Shimadzu, Japan), interfaced with a mass spectrometer (SYNAPT G2, Waters, UK) equipped with an electrospray-ionization source operated in the negative mode. Chromatographic separations were performed using a Thermo Hypersilgold C18 column (5 μm, 2.1 × 250 mm) using stepwise gradient elution with water (solvent A) and acetonitrile (solvent B) at a flow rate of 0.5 mL/min. The gradient program was 0–65 min 90% B; 65–150 min 90% B; 150 min 50% B. The injection volume was 20 μL. The confirmation ion transitions for quantification were *m*/*z* 621.5605→161.0162 for CK.

### 4.5. Determination of Antioxidant Effects

DPPH radical scavenging activity was determined based on the method of Blois [41] and Ratha et al. [42]. Briefly, 0.4 mM DPPH was prepared in absolute ethanol and 3.8 mL of the solution was added to each 0.2 mL of diluted sample. After 10 min, optical density was measured at 525 nm using UV/Vis Spectrophotometer (S22, Biochrom, UK). DPPH radical scavenging activity was expressed in the percentage of difference of extract sample compared to control.

ABTS radical scavenging activity was determined based on the method of Re et al. [43]. A 7 mM ABTS radical solution was mixed with a 140 mM potassium persulfate solution to prepare the ABTS solution, which was reacted for 12 h. The solution was diluted with absolute ethanol until the absorbance at 734 nm was 0.700 ± 0.02, and then it was used as the ABTS+ reagent. A 5 mL aliquot of ABTS+ reagent and 0.2 mL of sample solution were mixed for 6 min at room temperature. The absorbance of the final reacting solution was measured at 734 nm in a 96-well plate. ABTS radical scavenging activity was expressed in the percentage of difference of extract sample compared to control.

### 4.6. Cell Culture and Cytotoxicity Assay

RAW 264.7 murine macrophage cell lines were purchased from Korea Cell Line Bank (Seoul, Korea). The cytotoxicity was evaluated by MTT assay. The cells were maintained in DMEM supplemented with 10% FBS and 1% mixture of penicillin (10,000 U/mL) / streptomycin (10,000 μg/mL). Cytotoxic effects of PGE and CKE were assessed MTT assay [44]. The cells were cultured at a density of 5 × 10^4^ cells/ well in a 96-well and incubated for 24 h, in an atmosphere of 5% CO_2_. Samples of different concentrations of PGE and CKE were added and incubated for 3 h, and then stimulated with LPS (1 μg/mL) for 24 h. After discarding the medium, MTT solution (100 μL) was added to each well and incubated for 2 h at 37 °C, and then DMSO was added to dissolve the formazan dye crystals. The optical density of the reaction mixture was measured at 540 nm using an ELISA microplate reader (Epoch2, Bio Tek, Winooski, VT, USA). The cell viability was calculated as follows: Cell viability was expressed in percentage of absorbance difference at 540 nm of a test sample compared to control.

### 4.7. Determination of Anti-Inflammatory Effects

#### 4.7.1. Determination of NO Levels

The effects of PGE and CKE on NO levels were determined in RAW 264.7 cells. Cells were seeded in a 96-well microplate at a density of 5 × 10^4^ cells/well and treated with different concentrations of PGE and CKE for 3 h at 37 °C. Then 1 μg/mL of LPS was added to each well and incubated for 24 h. After incubation, 100 μL aliquot of each medium was transferred to a new well and 100 μL of Griess reagent was added to it. After 15 min, absorbance was measured at a wavelength of 540 nm using the ELISA microplate reader. The amount of nitrite present was calculated from a sodium nitrite standard curve.

#### 4.7.2. Determination of Pro-Inflammatory Cytokines

Levels of TNF-α, IL-1β and IL-6 were measured in RAW 264.7 cells to evaluate anti-inflammatory activity. Cell culture conditions were the same as those used for the NO assay. After incubation for 24 h, the concentrations of TNF-α, IL-1β and IL-6 in the cell media were determined using commercial ELISA kits, respectively, according to the manufacturer’s protocols. The absorbance was measured at a wavelength of 450 nm using an ELISA microplate reader.

### 4.8. Bleeding Assay

The bleeding experiment was approved by the Institutional Animal Experiments Local Ethics Committee of Biocenter of Gyeonggi Province (No. 2018-06-0004).

SPF ICR mouse was supplied from Orient bio Co. (Seongnam, Korea). The mice were maintained at room temperature (23 ± 3 °C), relative humidity 55 ± 15%, and they had free access to a commercial pellet diet and drinking water before experiments. The mice were randomly and equally divided into 5 groups each containing 6 mice; control/excipient group (distilled water), positive control group (aspirin 100 mg/kg/day), 3 test group (70, 100 and 150 mg/kg/day of CKE). The test samples were administered orally once a day for 7 days. The tail transection bleeding time was determined according to the method of Dejana et al. [45] and Lee and Kim [46]. The mouse tail was transected at 5 mm from the tip and it was immediately immersed into warmed (37 °C) saline. The bleeding time was determined as the time from the tail transection to the moment the bleed flow stopped. Bleeding time beyond 900 s was considered as cut-off time for the statistical analysis. The increase rate of bleeding time was calculated by an equation as below, where A is the bleeding time of the control group, B is the bleeding time of the test group.

$$\mathrm{Increase}\;\mathrm{rate}\;\left(\%\right)=\frac{B-A}{A}\times 100$$

In addition to bleeding time, bleeding volume was measured as a factor of antithrombotic effect. The blood volume was calculated using a calibration curve of an optical density value (550 nm) measured by adding a constant volume of blood to 12 mL of saline.

### 4.9. Statistical Analysis

Experiments were conducted using triplicate samples and were repeated three times. The values were expressed as mean ± standard deviation (or standard error of the mean in bleeding assay) and one-way analysis of variance (ANOVA) was carried out using SPSS software (version 22, SPSS Inc., Chicago, IL, USA). Duncan’s multiple range test was employed to test for significant differences between the treatments at *p* < 0.05.

## 5. Conclusions

It was estimated that ginseng sprouts could be an efficient and clean source of ginsenosides, in that they contained higher content of PPD type ginsenoside than that of 6-year-old ginseng roots and CK was converted effectively from the ginsenosides by enzymatic hydrolysis. CKE derived from ginseng sprouts showed higher anti-inflammatory effects than PGE in RAW 264.7 cells, reducing the levels of NO and pro-inflammatory cytokines while PGE showed higher antioxidant activity compared to CKE. From these results, it was presumed that the high content of CK in the CKE might play an important role in anti-inflammatory effects. In the in vivo test for antithrombotic effect, the CKE administered group showed increased effects on both bleeding time and bleeding volume than the control group. The increase of bleeding volume was relatively low compared to the aspirin administered group, which could be an advantage in treating thrombosis because excessive bleeding is undesirable. Ginseng sprout itself or the processed product such as CKE showed potential to be used for anti-inflammation and antithrombosis.

## Figures and Tables

**Table 1:** Total phenolic compound and crude saponin content in ginseng sprout and six-year-old ginseng root.

Test Group	Part	Phenolic Compound (mg/g dry wt.)	Saponin Content (%, *w*/*w*)	Ratio of Dry Weight (%, *w*/*w*)
Ginseng sprout	Leaf	26.32 ± 1.78 ^a^	34.41 ± 0.76 ^a^	20.44 ± 0.29 ^b^
	Stem	5.21 ± 2.02 ^c^	4.78 ± 0.29 ^d^	11.81 ± 0.15 ^c^
	Root	6.12 ± 0.93 ^c^	12.23 ± 0.51 ^c^	67.84 ± 0.24 ^a^
	Total	10.20 ± 1.51 ^b^	15.89 ± 0.71 ^b^	-
Six-year-old ginseng root	Root	6.54 ± 0.35 ^c^	11.67 ± 0.20 ^c^	-

Three independent experiments were carried out for each analysis. Result is expressed as a mean ± SD (*n* = 3). Values with the different letters in the column are significantly different by Duncan’s multiple range test (*p* < 0.05).

**Table 2:** Protopanaxadiol (PPD) type ginsenoside composition in ginseng sprout and 6-year-old ginseng root.

Test Group		Ginsenoside (mg/g Dry wt.)						
		Rb1	Rb2	Rc	Rd	F2	Rg3	PPD Total
Ginseng sprout	Leaf	9.56 ± 0.41 ^a^	5.54 ± 0.41 ^a^	9.43 ± 0.12 ^a^	7.3 ± 90.24 ^a^	9.45 ± 0.40 ^a^	ND	41.28 ± 0.22 ^a^
	Stem	3.01 ± 0.23 ^b^	1.34 ± 0.26 ^b^	1.04 ± 0.25 ^d^	0.63 ± 0.12 ^c^	2.92 ± 0.17 ^b^	ND	8.94 ± 0.21 ^c^
	Root	1.54 ± 0.14 ^c^	1.04 ± 0.43 ^b^	3.92 ± 0.33 ^b^	1.95 ± 0.06 ^bc^	2.08 ± 0.05 ^c^	ND	10.53 ± 31 ^bc^
	Total	3.32 ± 0.17 ^b^	1.98 ± 0.23 ^b^	4.68 ± 0.18 ^b^	2.86 ± 0.13 ^b^	3.66 ± 0.20 ^ab^	ND	16.64 ± 0.22 ^ab^
Six-year-old ginseng root		2.52 ± 0.45 ^b^	1.95 ± 0.14 ^b^	2.34 ± 0.32 ^c^	2.58 ± 0.63 ^b^	2.21 ± 0.10 ^c^	ND	11.6 ± 0.21 ^b^

ND: Not Detected. Three independent experiments were carried out for each analysis. Result expressed as a mean ± SD (*n* = 3). Values with the different letters in the column are significantly different by Duncan’s multiple range test (*p* < 0.05).

**Table 3:** Changes of PPD type ginsenosides during the enzymatic conversion process.

Ginsenoside	Ginsenoside Content (μg/mL)		
	Before Reaction	24 h after Reaction	48 h after Reaction
Rb1	102.51 ± 4.90	1.55 ± 0.02	ND
Rc	125.24 ± 1.92	89.85 ± 1.91	50.93 ± 0.41
Rb2	88.42 ± 5.52	ND	ND
Rd	95.43 ± 2.23	48.31 ± 0.73	0.3 ± 0.04
F2	97.58 ± 3.54	245.60 ± 5.03	33.6 ± 0.23
Rg3	ND	17.85 ± 0.60	53.5 ± 0.91
Compound K (CK)	ND	79.14 ± 0.95	235.71 ± 5.90
Total	509.01 ± 4.95	473.71 ± 1.90	373.21 ± 2.95

ND: Not Detected. Result expressed as a mean ± SD (*n* = 3).
